# COVID-19 Changed Prevalence, Disease Spectrum and Management Strategies of Ocular Trauma

**DOI:** 10.3389/fmed.2021.774493

**Published:** 2022-01-10

**Authors:** Haozhe Yu, Minhui Xu, Yue Zhao, Jingyi Li, Wenyu Wu, Yun Feng

**Affiliations:** ^1^Department of Ophthalmology, Peking University Third Hospital, Beijing, China; ^2^Institute of Medical Education, Peking University, Beijing, China

**Keywords:** COVID-19, ocular trauma, prevalence, management, disinfectants

## Abstract

The coronavirus disease 2019 (COVID-19) pandemic has significantly impacted the health of people around the world and has reshaped social behaviors and clinical practice. The purpose of this perspective is to provide epidemiologists and clinicians with information about how the spectrum of ocular trauma diseases changed, as well as to optimize management for improving patient prognosis during this crisis. Analysis of current studies revealed that the prevalence of eye trauma decreased overall, with a trend of delayed medical treatment during the COVID-19 era. Irregular epidemic prevention and control measures, unprotected home activities, and unusual mental states are the main causes of ocular trauma. Strategies for reducing morbidity are also discussed, including popularizing the use norms of prevention and control supplies, taking heed to the safety of family activities, highlighting the special status of child protection, and paying attention to previous case data to implement region-specific precautions. The procedure of ophthalmological emergency and outpatient management should also be optimized, and mental health should be emphasized during this pandemic.

## Introduction

The coronavirus disease 2019 (COVID-19) pandemic has demonstrated wide-ranging effects on the society and the healthcare system. Many countries have executed strong “Shelter-in-Place” policies to curb its spread. However, such measures have significantly impacted the mental preference and behavioral pattern of individuals, which has led to further changes in the disease spectrum (1, 2). It has been proven that during this pandemic, there exists an increasing trend in interpersonal violence-related trauma and traffic-related trauma due to anxiety, irritability, and cancellation of public transportation (3–6). The pandemic has also significantly disrupted scheduled medical activities, through the redeployment of more healthcare resources to COVID-19 patients and addition of COVID-19 screening for infection control before treatment (7, 8). In addition, patients grew more reluctant to seek health care, for fear of nosocomial infection, and even delay treatment (9).

Ocular trauma is an acute and severe disease in ophthalmology, and is an important cause of monocular blindness, which has a huge socioeconomic burden (10–12). Work-related injuries such as intraocular foreign body (IOFB) caused by machine elements and corneal burn caused by chemical reagents are the most common mechanisms of ocular trauma, followed by the tools used in the home and fireworks in festival activities (13–15). Seasonal trends in the incidence of ocular trauma were also observed, being higher in spring and summer, owing to the increase in outdoor activities (16, 17). Prompt diagnosis with a high standard of emergency management can provide patients with better visual outcomes and prognoses (18, 19). Considering the paradigm shift in all aspects of life and healthcare during the ongoing COVID-19 pandemic, we assumed that the disease spectrum and care paths of ocular trauma would change. This perspective narratively reviewed the COVID-19-related changes in the morbidity and disease spectrum of ocular trauma and discussed its potential prevention and control measures with the aim of optimizing the emergent treatment and management in the COVID era.

## Methods

**Figure 1:**
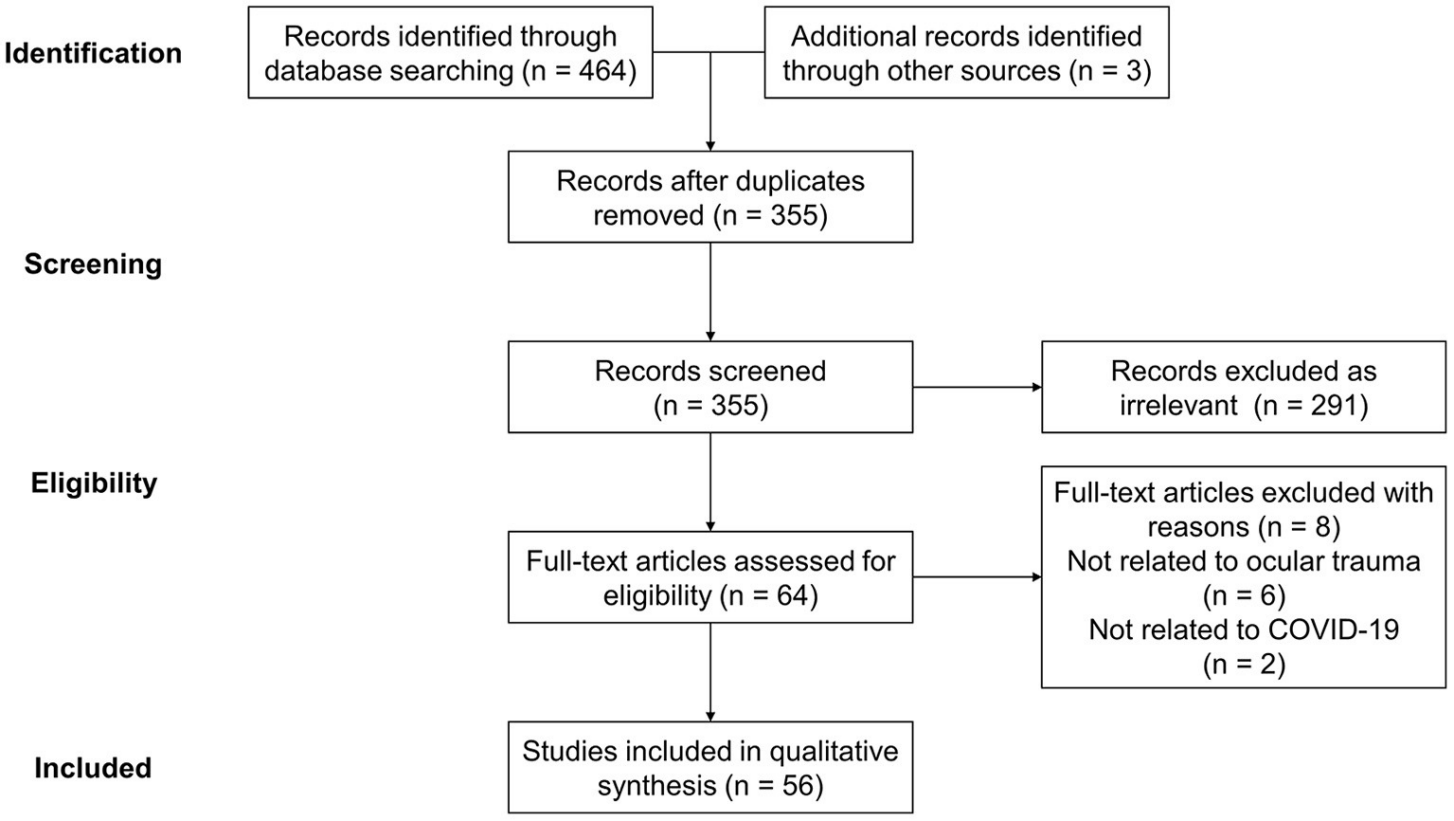
Flow diagram of literature searching and selecting.

The impact of the COVID-19 pandemic on the morbidity and management of ocular trauma were determined through literature search in the databases of Web of Science, Embase, Cochrane and PubMed, and presented as a narrative review owing to the novelty of related studies (20). The search terms were identified as Birmingham Eye Trauma Terminology, “Coronavirus,” “COVID-19,” “SARS-Cov-2,” “Ocular trauma,” “Eye injury” and their variations. The search was conducted in November 2021, and all the references of included articles were reviewed to prevent missing associated research. All literatures fitting the theme were included regardless of their article types, and the searching and screening were performed independently by three reviewers (MX, HY, and YZ) while the disagreements were resolved by a fourth investigator (YF). A total of 467 records were retrieved, of which 56 were included through title and abstract analysis and further full-text content assessment for eligibility (Figure 1).

## Results

### Impact on Morbidity and Patient Characteristics

Several studies have reported an overall decreasing trend in the morbidity of ocular trauma, and the number of emergency eye services has also decreased during the COVID-19 era, especially for traffic and work-related trauma. Meanwhile, interpersonal violence-related injuries were at a relatively high level (21–27). A case-control study in the United Kingdom noted that within the first month after the national lockdown order was issued, the total number of ophthalmological emergency visits decreased by 53.1% compared with the same period the year before. Likewise, the ocular trauma-related presentations decreased by 43.9%, which could be mainly attributed to fewer outdoor activities due to movement restrictions and social distancing (28). A similar finding was reported in India, where the incidence decreased by 58.5% during the lockdown. However, upon the lifting of the lockdown, the number of emergency visits due to ocular trauma increased monthly (29).

During quarantine, men were still the most frequent victims, and in some studies, the proportion of male patients presenting with ocular trauma has increased further (30, 31). There was a disputable change in the mean age of patients with ocular trauma before and after the outbreak, which may have resulted from the differences in the age composition of the population and economic structure between cities. A review in the United States reported a 2.4-year increase in the average age of patients, and in Italy, a 2.6-year increase was also reported (31, 32). However, there was a significant reduction in the average age of patients in India, with a decreased proportion of patients aged >50 years and an increased proportion of patients aged between 16 and 50 years (29, 33, 34). Moreover, the prevalence of serious ocular trauma, such as eyelid laceration, open globe injury, and traumatic retinal detachment, was reported to be higher during the COVID-19 era, which could be attributed to the changes in the behavioral pattern of individuals and reduced likelihood of patients with mild trauma seeking healthcare (35). The widespread use of online ophthalmic consultation services during the outbreak further confirmed this view (28). In addition, there was a trend of delay in emergency department visits for ocular trauma, and Stedman et al. (36) revealed that the delay between onset and visit increased from 0.33 days to 1.1 days before and after COVID-19, respectively.

### Impact on Disease Spectrum

Table 1 summarizes the changes in the mechanisms of ocular trauma during the COVID-19 pandemic. There was an increase in the ocular trauma associated with the prevention and control of virus transmission during the pandemic, mainly including keratoconjunctivitis photoelectrica caused by ultraviolet (UV) disinfection, and chemical ocular injuries caused by disinfection products such as hand sanitizers. Keratoconjunctivitis photoelectrica has been a typical occupational injury since before COVID-19, occurring mostly in industries with high UV exposure such as electric welding. A large number of non-occupational cases appeared after the outbreak, which nearly doubled compared with the same period the year before (37). With the general use of UV-C rays to prevent airborne virus transmission, acute exposure to high-dose UV radiation arising from proximity to the light source, and lack of self-protection has emerged as a key risk factor that could induce topical inflammation and cause damage to corneal and conjunctival epithelial cells (57, 58). Symptoms usually appear a few hours after exposure, from the earliest gritty sensation to ocular pain or discomfort, conjunctival congestion, photophobia, and tearing (59). Fortunately, most cases were mild with a good visual prognosis, requiring only supportive treatment to promote epithelial repair, resist infection, and prevent scarring (60). Leung and Ko (39) reported that three adults developed photophobia, ocular pain, and decreased visual acuity after direct exposure to a UV germicidal lamp; no corneal epithelial staining was found, and the symptoms were relieved after receiving lubricating eye drops. Sengillo et al. (40) reported seven patients presenting with ocular pain, conjunctival congestion, and punctiform corneal epithelial erosion following overexposure to UV. They all recovered well after treatment with artificial tears, topical antibiotics, or steroids.

**Table 1:** Changes in disease spectrum of ocular trauma before and after COVID-19.

**Mechanism**	**Changes in ocular trauma cases per mechanism**
Irregular epidemic prevention and control measures	• Cases associated with UV overexposure were tripled in China (37)
	• 109 cases of photokeratitis in a hospital in China within 8 weeks after the outbreak, increased nearly four times (38)
	• 3 and 7 cases of UV-photokeratitis were reported, respectively (39, 40)
	• In Croatia, the consultations related to exposure to disinfectants and hand sanitizers were doubled and ten-fold respectively (41). Some relevant cases were reported (42)
	• A seven-fold increase in pediatric cases related to hand sanitizers in France (43)
	• 9 pediatric cases related to hand sanitizers in Israel, which was not seen last year (44)
	• 2 pediatric cases of toxic keratopathy related to hand sanitizers (45)
	• 9 cases related to masks in China (46)
Home accidents	• A 42.4% increase in the cases occurring at home in India, and 5.5% in the U.S. (29, 32)
	• 23 cases occurring at home per week, in comparison to 10 in 2019 (47)
	• 4 cases related to home accidents in Jorden (48)
	• 4.6% and 2.2% increase in cases related to home activities and gardening in Italy, respectively (31)
	• A 28% increase in cases related to home improvement in the U.S. (35)
	• Many cases related to gardening was reported (49, 50)
	• DIY-related cases were three-fold compared to normal days (36)
	• 2 cases related to “bow and row” in India (51)
	• 2 cases secondary to exercise resistance band were reported respectively (52, 53)
Outdoor accidents	• A 37.9% decrease in the cases occurring in the workplace in India, and 4.3% in the U.S., (29, 32)
	• 5.5 and 3% decrease in cases related to falls and outdoor sports in Italy, respectively (31)
Interpersonal violence	• A 5% increase in call-outs and 75% increase in Internet searches related to intimate partner violence, even as overall crime dropped 40% in Australia; a 32–36% increase in intimate partner violence complaints in France, and 21–35% in the U.S. after quarantine measures (54)
	• 45% of intimate partner violence injuries involved eyes (55)
	• A 5% increase in the cases related to intimate partner violence in North India (33)
	• Cases related to pepper spray in protest march against COVID-19 restrictions (56)
	• A 1.2% increase in the cases related to interpersonal violence (31)

Regular use of disinfectants and hand sanitizers is also recommended for the prevention of COVID-19, although the hypochlorous acid and alcohol compounds contained therein could lead to concentration-dependent corneal and limbal toxicity, including superficial epithelial denudation, topical inflammation, and stem cell deficiency (61, 62). Both direct and indirect contact through the volatilization of disinfectant components in the air could induce ocular inflammation, especially in patients with chronic allergies, compromised ocular surfaces, and previous ocular surface diseases like dry eyes (63). Babić et al. (41) reported that exposure to disinfectants and hand sanitizers during the COVID-19 outbreak was, respectively, twice and 10 times that of the same period the previous year in Croatia. Adults were at high risk of exposure to disinfectants, while children were at high risk of exposure to hand sanitizers. In France, it was also found that the incidence of ocular trauma caused by alcohol-based hand sanitizer in children increased by seven times compared with the same period in 2019, with nearly 38% of patients with corneal ulcers involving over 50% of the ocular surface and nearly 13% of patients requiring amniotic membrane transplantation (43). Chemical ocular injuries caused by hand sanitizers in children usually have a good prognosis after treatment. Wasser et al. (44) reviewed nine cases of ocular chemical injuries caused by hand disinfectants and indicated that after washing and topical antibiotics, hormones, and lubricants, 67% of the children were discharged and the others with more severe ocular surface injuries involving over 85% cornea and 30–75% conjunctiva received an average of 7 days of hospitalization and were healed within 19 days, with good visual recovery and no irreversible damage. Yangzes et al. (45) also reported two cases of toxic keratopathy in children caused by hand disinfectants during the COVID-19 outbreak. One patient presented with a large central corneal defect combined with conjunctival edema and ischemia, and the other presented with superficial punctate keratopathy. After receiving antibiotics, glucocorticoids, topical lubricants, as well as additional antibiotics and vitamin C orally, the ocular surface of both patients recovered well.

Given the impact of the pandemic and related containment strategies, the lifestyle and workstyle of individuals underwent a great shift, and correspondingly, the prevalence of ocular trauma occurring in domestic settings was significantly higher than in the workplace (64–66). Pellegrini et al. (31) reported that during quarantine, the proportion of ocular trauma caused by falls and sports decreased significantly by ~5.5 and 3%, respectively. On the other hand, the percentage of ocular trauma caused by home activities and gardening, respectively, increased from 12.4 and 8.5% in 2019 to 17.0 and 10.7%. The surge of ocular trauma occurring at home could be mainly attributed to gardening, do-it-yourself (DIY) activities, home improvement, and exercise. Although most cases were mild, there were also open globe injuries with a high risk of blindness, constituting the majority of severe ocular injuries during the COVID-19 era. Several studies have reported a variety of types of ocular trauma caused by gardening, including epithelial defects or fungal keratitis caused by leaf scratches, corneal or conjunctival burns caused by improper use of herbicides, and open-global injuries or perieyelid injuries caused by splashes of plant debris or stones thrown by lawnmowers (67). A case of ocular trauma in a 58-year-old man caused by gardening during this COVID-19 period was reported by Nocini et al. (50), in which a nail thrown by the lawnmower pierced his left upper eyelid and penetrated the superior rectus and lateral rectus muscles, leading to restricted globe movement. Sputtering particles from DIY and amateur home improvement activities are also the leading causes of serious ocular trauma.

A retrospective cohort analysis in the United Kingdom noted that the number of serious ocular traumas, including rupture, lid lacerations, and IOFB, was three times the average over the past 5 years, with a 400% increase in hospital admissions due to DIY activities after the outbreak (36). Wu et al. (35) also reported a nearly 30% increase in the proportion of serious ocular trauma caused by home improvement during the COVID-19 pandemic. Exercise resistance bands, mainly used for strength training and a good substitute for gymnasiums, were the most reported sources of exercise-related ocular trauma occurring at home during COVID-19. Sibley et al. (52) first reported two cases of ocular trauma caused by exercise resistance bands during the lockdown in the United Kingdom. A 41-year-old woman had a 0.8-mm hyphema in the right eye, 1.0-mm in the left eye, and peripheral commotio retinae after being hit in both eyes. The other case was a 19-year-old young man presenting with red blood cell deposition on the inferior corneal endothelium and commotio retinae in the right eye, and a 1-mm hyphema, vitreous and intraretinal hemorrhages, and retinal break in the left eye. Both patients presented with binocular involvement and asymmetrical injuries, with the left eye being more severe than the right eye. Al-Khersan et al. (53) further reviewed 11 cases of ocular trauma caused by exercise resistance bands during the COVID-19 era and reported a wide range of subsequent ocular injuries, including corneal epithelial defects, iris defects, iritis, hyphema, vitreous hemorrhage, retinal tear, commotio retinae, macular hole, and permanent vision loss.

Multiple studies have indicated that the prevalence of ocular trauma associated with interpersonal violence is high during this pandemic, which could be mainly attributed to mental anxiety and depression arising from movement restrictions (35, 68). Sissoko et al. (68) reported a striking increase in ocular trauma related to interpersonal conflicts, especially quarrels and protest marches after the stay-at-home order was initiated, with corneal and maxillofacial injuries as major symptoms. Intimate partner violence also emerged as a leading cause of ocular trauma, which was severe and worsened with scleral laceration, vision loss, and ultimately enucleation (69). Previous studies have illustrated a 27% increase in domestic violence reports received by police in the United States since the travel restriction orders were issued, of which the proportion of serious injuries nearly doubled, suggesting higher severity (70, 71). In addition, the incidence of pediatric ocular trauma increased markedly, and children aged 13–18 years became a high-risk group for open-globe injuries during the pandemic rather than preschoolers (72). Apart from the aforementioned risk factors of epidemic prevention, home activities, and interpersonal violence, children's imitation of risky behaviors on entertainment programs could also result in ocular trauma. Bapaye et al. (51) reported that within 4 days after the broadcast of an Indian TV program, two Indian children suffered ocular trauma while imitating the “archery” part of the program. The first one presented with epithelial defects after being hit by a rubber arrow, and the other developed traumatic cataract and endophthalmitis after an arrow made from a broom stick shot the cornea and entered into the eye. However, related cases were rare prior to the COVID-19 outbreak.

## Discussion

Despite a decreased incidence of ocular trauma during the COVID-19 pandemic, the proportion of serious cases has increased, with a significant shift in risk and pathogenesis. Fortunately, most ocular traumas are preventable, which underscores the need for targeted precautions. First, since products against viruses, including disinfectants, hand sanitizers, and UV germicidal lamps, have become widely available and have led to an increased incidence of related ocular trauma, it is necessary for the media and local businesses to clarify the potential hazards to the public and popularize the use norms of these (37, 41). Further, wearing goggles or sports glasses is central to the prevention of ocular trauma due to home activities, such as gardening, DIY activities, and exercise, although it remains unpopular to date (53, 73). As individuals were confined to their homes and engaged in more home activities during the lockdown, great importance should be attached to the education on the risk of ocular trauma and the promotion of associated protective measures. In addition to the goggles, it is also effective to encourage the media to broadcast home leisure activities and exercise instruction programs.

Further, parents should strengthen the supervision and protection of children, especially in the use of prevention and control supplies and potentially dangerous items, to prevent accidental ocular trauma due to mental immaturity (45, 72). Moreover, given the temporal and spatial specificity of ocular trauma, it is critical to collate previous cases and summarize the region-specific pathogenic characteristics of ocular trauma before formulating regional preventive strategies. All preventive measures contribute to reducing the incidence and severity of ocular trauma and the burden on the national healthcare system.

Since ocular trauma often leads to poor visual prognosis and blindness, timely treatment is important. The lag between onset and presentation was common during the lockdown, which may further worsen the symptoms, such as a large corneal ulcer (36, 47). Reduced access to healthcare may be a key factor in the lag. Thus, it is recommended that the healthcare system optimizes the procedure of ophthalmological emergency and outpatient care to ensure timely treatment of ocular trauma despite limited medical resources. Remote ophthalmic services may be an effective strategy to provide specialized health care in time and have been implemented in many countries. This could tilt medical resources toward emergency patients with serious ocular trauma, and also decrease the risk of viral infection and transmission by avoiding visits of non-emergency patients (28, 29).

Widespread psychological problems, such as anxiety, depression, and distrust caused by social distancing during the pandemic, should be taken seriously, as they are considered to be the main reasons for the surge of ocular trauma cases associated with interpersonal conflicts (35). Home activities and online communication are both effective in alleviating panic and anxiety and maintaining mental health during quarantine. Moreover, it is necessary to promptly disclose information associated with the pandemic, as misinformation distributed by social media could trigger a cascade of fear and anxiety among the public (74). On the other hand, the same psychological problem also existed among medics, partly owing to a particularly high risk of infection. Authorities concerned should emphasize the mental health of medics and provide timely psychological counseling through online psychological consultation or other methods.

## Data Availability Statement

The original contributions presented in the study are included in the article/supplementary material, further inquiries can be directed to the corresponding author.

## Author Contributions

HY and MX conceived and designed the entire study and wrote the initial draft of the manuscript. All authors contributed to the article and approved the submitted version.

## Funding

This study was supported by National Natural Science Foundation of China Grants (Nos. 81700799 and 82070926).

## Conflict of Interest

The authors declare that the research was conducted in the absence of any commercial or financial relationships that could be construed as a potential conflict of interest.

## Publisher's Note

All claims expressed in this article are solely those of the authors and do not necessarily represent those of their affiliated organizations, or those of the publisher, the editors and the reviewers. Any product that may be evaluated in this article, or claim that may be made by its manufacturer, is not guaranteed or endorsed by the publisher.
